# Effects of physical exercise interventions on multidimensional health outcomes in patients with substance use disorders: a network meta-analysis

**DOI:** 10.3389/fpsyt.2025.1732663

**Published:** 2026-01-14

**Authors:** Taihu Liu, Zhiqiang Chen, Kerun Gao

**Affiliations:** 1 Affiliated Mental Health Center & Hangzhou Seventh People’s Hospital, Zhejiang University School of Medicine, Hangzhou, China; 2 School of Civil Engineering, Xinyang University, Xinyang, China; 3 College of Physical Education, Kashi University, Kashi, China

**Keywords:** physical exercise, substance use disorders, multidimensional health outcomes, intervention effects, network meta-analysis

## Abstract

**Objective:**

This study investigated the intervention effects of different physical exercise programs on multidimensional health outcomes in patients with substance use disorders. Through systematic categorization of intervention types, duration, frequency, and outcome indicators, this research aimed to provide evidence-based exercise prescriptions for rehabilitation treatment of patients with substance use disorders.

**Methods:**

A systematic search was conducted across six databases: PubMed, Web of Science, Cochrane Library, EMBASE, SCOPUS, and ScienceDirect, retrieving 11,689 articles related to substance use disorders. The search period was limited from database inception to July 1, 2025. Data extraction and quality assessment were performed using Review Manager 5.4 software, ultimately including 33 articles meeting inclusion criteria, encompassing 2,922 participants. Subsequently, network meta-analysis was conducted using Stata 16.0, with results presented as standardized mean differences (SMD) and 95% confidence intervals (CI).

**Results:**

A total of 33 randomized controlled trial articles were included, comprising 57 randomized controlled trials with 2,922 subjects. Network meta-analysis results demonstrated that aerobic exercise and mind-body exercise exhibited significantly different advantages: aerobic exercise performed optimally in physiological health indicators (SUCRA = 0.874), followed by substance use outcomes (SUCRA = 0.468) and sleep quality (SUCRA = 0.446); mind-body exercise showed the greatest advantage in improving sleep quality (SUCRA = 0.884), with cognitive function (SUCRA = 0.608) and mental health (SUCRA = 0.588) ranking second and third, respectively. In pairwise comparisons, substance use outcomes in the mind-body exercise network showed significant advantages compared to sleep quality (effect size 44.58, 95% CI: 3.30-85.85), while all comparisons in the aerobic exercise network did not reach statistical significance.

**Conclusion:**

Different types of exercise exert therapeutic effects in their respective advantage domains through unique molecular biological mechanisms. Aerobic exercise primarily improves physiological health indicators through AMPK signaling pathways and anti-inflammatory mechanisms, while mind-body exercise optimizes sleep quality and cognitive function through HPA axis regulation and GABA receptor upregulation. These findings provide important evidence-based medical evidence for developing personalized exercise prescriptions for substance use disorders, contributing to the establishment of evidence-based rehabilitation treatment guidelines.

**Systematic review registration:**

https://www.crd.york.ac.uk/PROSPERO/, identifier CRD420251144089.

## 1 Introduction

Substance use disorders represent one of the most serious public health challenges globally, encompassing the abuse and dependence on alcohol, drugs, and other psychoactive substances. These disorders are characterized by compulsive use, increased tolerance, and withdrawal symptoms, severely impacting patients’ physical and mental health as well as social functioning (1). According to the latest statistics from the United Nations Office on Drugs and Crime’s 2024 World Drug Report, approximately 296 million people worldwide used illicit drugs in the past year, with about 39.4 million suffering from drug use disorders—an 11% increase compared to 2021, reflecting the persistent deterioration of global substance use problems (2). The World Health Organization’s 2024 Global Health Observatory report indicates that substance use disorders contribute 163 million disability-adjusted life years to the global disease burden, accounting for 6.1% of the total global disease burden, presenting a major challenge for governments and health departments worldwide (3). Furthermore, patients with substance use disorders not only face physiological dependence issues but also commonly experience comorbid mental health problems such as depression and anxiety. Research demonstrates that approximately 50-70% of patients have comorbid psychiatric disorders, while also exhibiting significant deficits in cognitive function, sleep quality, and social functioning (4, 5). The complexity of this multidimensional health impairment makes single intervention approaches often inadequate for achieving optimal therapeutic outcomes, necessitating the development of more comprehensive and effective treatment strategies.

Traditional substance use disorder treatment primarily relies on pharmacological substitution therapy and psychological behavioral interventions, which, while demonstrating certain efficacy in acute symptom management, still present numerous limitations. Although pharmacological treatments can alleviate withdrawal symptoms and reduce craving, long-term use carries risks of developing new dependencies, tolerance, and adverse reactions, while also being costly and unable to comprehensively improve patients’ multidimensional health status (6, 7). The Substance Abuse and Mental Health Services Administration (SAMHSA) 2025 treatment guidelines emphasize that traditional pharmacological treatments still have relapse rates of 40-60%, highlighting the limitations of existing treatment models (8). While psychological behavioral interventions play important roles in cognitive restructuring and behavioral change, their effectiveness is often influenced by multiple factors including patient adherence, therapist competency, and treatment environment, with limited long-term maintenance effects (9). Therefore, exploring safe, effective, and cost-efficient adjunctive treatment approaches has become an important direction in current addiction medicine research.

In recent years, physical exercise as a non-pharmacological intervention has gained considerable attention in substance use disorder treatment, with its theoretical foundation rooted in the intersection of exercise neurobiology and addiction neuroscience research. Lynch (10) proposed the exercise-addiction neurobiology hypothesis, suggesting that exercise can exert therapeutic effects by modulating reward systems, improving neuroplasticity, and promoting neurotransmitter balance, providing a solid scientific foundation for exercise interventions (10). In 2024, the World Health Organization published the “Comprehensive Guidelines for Addiction Treatment,” which for the first time listed physical exercise as a recommended adjunctive treatment for substance use disorders, emphasizing the important role of exercise interventions in comprehensive treatment strategies (3). Compared to traditional treatment methods, physical exercise offers multiple advantages: low cost, minimal side effects, good adherence, high accessibility, and the ability to simultaneously improve patients’ physical health, psychological state, cognitive function, and social adaptation capabilities (11, 12). Additionally, exercise can provide healthy alternative activities, helping patients rebuild positive lifestyles and social support networks, which is crucial for long-term recovery.

Current research indicates that different types of physical exercise may exert therapeutic effects through distinct molecular biological mechanisms, presenting unique treatment approaches. Based on preclinical and neurobiological studies, aerobic exercise may achieve therapeutic effects through potential mechanisms including activation of the dopaminergic reward system, promotion of endorphin and brain-derived neurotrophic factor release, and improvement of cardiovascular function and metabolic status, though the direct causal relationships in clinical populations require further validation (13, 14). Greenwood et al. (15) further discovered that aerobic exercise can reshape the prefrontal cortex-striatum circuit, enhancing cognitive control abilities and thereby reducing impulsive substance use behaviors (15). Mind-body exercises such as tai chi and yoga primarily produce therapeutic effects by modulating the hypothalamic-pituitary-adrenal axis, enhancing parasympathetic nervous activity, improving sleep quality, and promoting mindfulness awareness (16, 17). These differential mechanisms of action suggest that different types of exercise may have respective advantages in various health dimensions, but there is currently a lack of systematic comparative studies to clarify the relative effects and applications of different exercise modalities.

Although multiple studies have explored the intervention effects of physical exercise on substance use disorders, existing systematic reviews and meta-analyses have obvious limitations. First, most studies focus on single types of physical exercise or specific outcome indicators, lacking direct comparisons of multidimensional effects between different exercise programs (18, 19). Second, traditional pairwise meta-analyses can only compare two interventions, making it impossible to achieve simultaneous comparison and ranking of multiple intervention programs, limiting the identification of optimal intervention strategies. Third, existing research predominantly focuses on single indicators such as substance use frequency or craving severity, neglecting patients’ comprehensive improvement needs across multiple dimensions including mental health, cognitive function, physical health, and sleep quality. Network meta-analysis can integrate direct and indirect comparison evidence, enabling simultaneous evaluation and ranking of multiple interventions, overcoming the limitations of traditional meta-analyses and providing more comprehensive and reliable evidence support for developing personalized treatment programs (20).

Based on this foundation, this study employs network meta-analysis methodology to systematically compare, for the first time, the intervention effects of aerobic exercise and mind-body exercise on multidimensional health outcomes in patients with substance use disorders. By integrating recent high-quality randomized controlled trials, we comprehensively evaluate the intervention effects of different exercise programs across five key dimensions: substance use outcomes, mental health, cognitive function, physical health, and sleep quality. Using the Surface Under the Cumulative Ranking Curve (SUCRA) method to rank various interventions and revealing relative effects between different exercise types through pairwise comparison analysis, this study aims to elucidate the relative advantages and potential molecular mechanisms of different exercise types. The goal is to provide high-quality evidence-based medical evidence for developing personalized exercise prescriptions for substance use disorders, promoting the application and development of precision rehabilitation concepts in addiction medicine, and ultimately improving patients’ multidimensional health status and quality of life.

## 2 Research methods

### 2.1 Literature search strategy

This systematic review and meta-analysis protocol was registered in the PROSPERO database (registration number: CRD420251144089). This study followed the Preferred Reporting Items for Systematic Reviews and Meta-Analyses of Network Meta-Analyses (PRISMA-NMA) guidelines for literature searching (21). Computer-based searches were conducted across six databases: PubMed, Web of Science, Cochrane Library, EMBASE, SCOPUS, and ScienceDirect, with search periods limited from database inception to July 1, 2025. A total of 11,689 articles related to substance use disorders were retrieved. Data extraction and quality assessment were performed using Review Manager 5.4 software, with 33 articles meeting inclusion criteria ultimately selected, encompassing 2,922 participants. Subsequently, network meta-analysis was conducted using Stata 16.0, with results presented as standardized mean differences (SMD) and 95% confidence intervals (CI).

The English search strategy combined Medical Subject Headings (MeSH) terms with free-text terms: ((“physical activity” OR “exercise” OR “physical training” OR “sport” OR “aerobic exercise” OR “mind-body exercise” OR “yoga” OR “tai chi”) AND (“substance use disorder” OR “drug addiction” OR “alcohol dependence” OR “substance abuse” OR “addiction”) AND (“randomized controlled trial” OR “RCT”)), with reference to the Medical Subject Headings vocabulary to standardize search term combinations. Synonyms were expanded to improve search recall rates. Chinese databases employed combined Chinese search strategies using subject headings and free-text terms.

Additionally, to further expand literature sources, manual screening of reference lists from all relevant meta-analyses and systematic reviews was conducted to supplement studies that met criteria but were not captured in the initial search (22). For different databases, researchers conducted preliminary literature screening to select studies meeting inclusion criteria. To ensure screening accuracy, all initially screened literature underwent independent full-text screening by two reviewers twice, with disagreements resolved through consultation with a third reviewer until consensus was reached. All included studies strictly adhered to quality assessment procedures, with two researchers independently conducting bias risk assessment for each randomized controlled trial according to the Cochrane Collaboration risk assessment framework (23). The two researchers cross-checked assessment results, with disagreements resolved through third-party evaluation. The data extraction process included detailed definitions for each variable: demographic characteristics including sample size, mean age, and gender composition; intervention protocols including specific exercise types, exercise intensity levels, weekly frequency, single session duration, and total intervention period; multidimensional health outcome assessments using relevant rating scales or indicators. Finally, the aforementioned demographic variables, intervention variables, and health outcome assessment variables were organized and summarized in data extraction tables.

### 2.2 Data and methods

#### 2.2.1 Inclusion criteria

1. Study participants were patients aged 18 years and older diagnosed with substance use disorders, including alcohol dependence, drug dependence, and other types of substance use disorders, meeting DSM-5 or ICD-10 diagnostic criteria.
2. Interventions consisted of mind-body exercises (such as tai chi, yoga, qigong) or aerobic exercises (such as jogging, brisk walking, cycling) in the experimental group, with control groups receiving either routine treatment (standard medical care including pharmacological and psychological interventions without exercise components) or waitlist control (delayed intervention after study completion).
3. Study design was randomized controlled trials, requiring specific descriptions of randomization methods, appropriate allocation concealment, and reporting of dropouts and loss to follow-up (24).
4. Primary outcome measures included substance use outcomes (abstinence/relapse rates, frequency of use, craving severity) and mental health outcomes (depression and anxiety symptoms), with corresponding measurement tools including TLFB, OCDS, VAS, HAMD, BDI, SDS, HAM-A, STAI, etc. Secondary outcome measures included cognitive function (Stroop, Go/No-Go, WCST, Digit Span, etc.), physiological health indicators (VO_2_max, 6MWT, HRV, etc.), and sleep quality (PSQI, Actigraphy, etc.).
5. Studies reporting sample sizes for each group and means and standard deviations for each outcome measure, as well as detailed statistical data including 95% confidence intervals and effect sizes that allow for network meta-analysis.

#### 2.2.2 Exclusion criteria

1. Study participants under 18 years of age, not involving patients with substance use disorders, or having unclear diagnostic criteria.
2. Interventions not including aerobic or mind-body exercise programs, or having unclear exercise protocol descriptions.
3. Presence of serious acute physical diseases or severe psychiatric disorder history that significantly affects physical and mental health.
4. Study design was not randomized controlled trial, or randomization methods were unclear, without appropriate allocation concealment (25).
5. Outcome measures did not include standardized assessment scales or used self-developed non-standardized assessment scales.
6. Unable to obtain complete analyzable data, such as sample sizes, means, and standard deviations for each group.
7. Duplicate data publication or extremely poor study quality.
8. Participants had serious adherence issues or substantial mid-study dropout problems.

### 2.3 Literature screening and data extraction

Two researchers independently reviewed literature and extracted data according to inclusion and exclusion criteria (26). Disagreements or inconsistent information between researchers were resolved through discussion with a third researcher. The following data were extracted from included randomized controlled trials: first author name; publication year; country; sample size (treatment group/control group); mean age; exercise intervention characteristics (content, duration, frequency, session length); outcome indicator types; effect sizes with standard errors and 95% CI; means and standard deviations for control and intervention group variables. The studies encompassed different types of physical exercise interventions and multidimensional health outcome indicators.

### 2.4 Quality assessment of included studies

Quality assessment of included RCT literature was conducted using Review Manager 5.4 provided by Cochrane (23). Assessment content included random sequence generation, allocation concealment, blinding of participants and personnel, blinding of outcome assessors, completeness of outcome data, selective reporting, and other sources of bias. “+” was rated as low risk of bias, “?” represented unclear risk, and “-” represented high risk of bias. Studies receiving 6-7 “+” ratings were classified as showing low risk of bias, 4-5 “+” indicated moderate risk of bias, and 4 or fewer “+” indicated high risk of bias (27).

### 2.5 Statistical analysis

This study employed frequentist network meta-analysis methodology using Stata 16.0 software for statistical analysis (28). Considering differences in sample sizes across studies, sample size-weighted means were used to calculate pooled standard deviations to improve estimation precision. Standardized mean difference (SMD) was used as the effect size indicator. Based on the network structure characteristics of this study, physical exercise interventions were categorized into mind-body exercise and aerobic exercise, with separate independent networks constructed for analysis.

Heterogeneity among included studies was assessed using Q-test and I² statistics (29, 30). Node-splitting models and global inconsistency tests were used to evaluate network consistency. The Q-test was calculated based on weighted sum of squares of effect sizes from individual studies; when the Q value exceeded degrees of freedom, significant heterogeneity among study effect sizes was indicated. The I² statistic directly reflected the degree of inconsistency among study effect sizes, comprehensively assessing heterogeneity status of included studies and serving as the basis for subsequent use of random-effects models for meta-analysis. I² values of 25%, 50%, and 75% represented low, moderate, and high degrees of heterogeneity, respectively.

Following heterogeneity testing, this study employed random-effects models to calculate weighted means of individual study effect sizes, obtaining final pooled effect sizes (31). Pooled effect size results reduced random errors from individual studies to some extent, enabling reasonable estimation of overall effect values and more accurate assessment of the magnitude of physical exercise intervention effects on multidimensional health outcomes in patients with substance use disorders. Weighted means comprehensively considered different weights assigned based on factors such as sample sizes and variances from individual studies, enabling more accurate and reliable pooled effect sizes.

Surface Under the Cumulative Ranking Curve (SUCRA) values were used to rank the effectiveness of different interventions, with higher SUCRA values indicating higher probability of being the best treatment (32). Publication bias was assessed using funnel plots and Egger’s test (33). All statistical tests used two-sided testing, with P<0.05 considered statistically significant.

## 3 Results

### 3.1 Literature screening process and results

**Figure 1:**
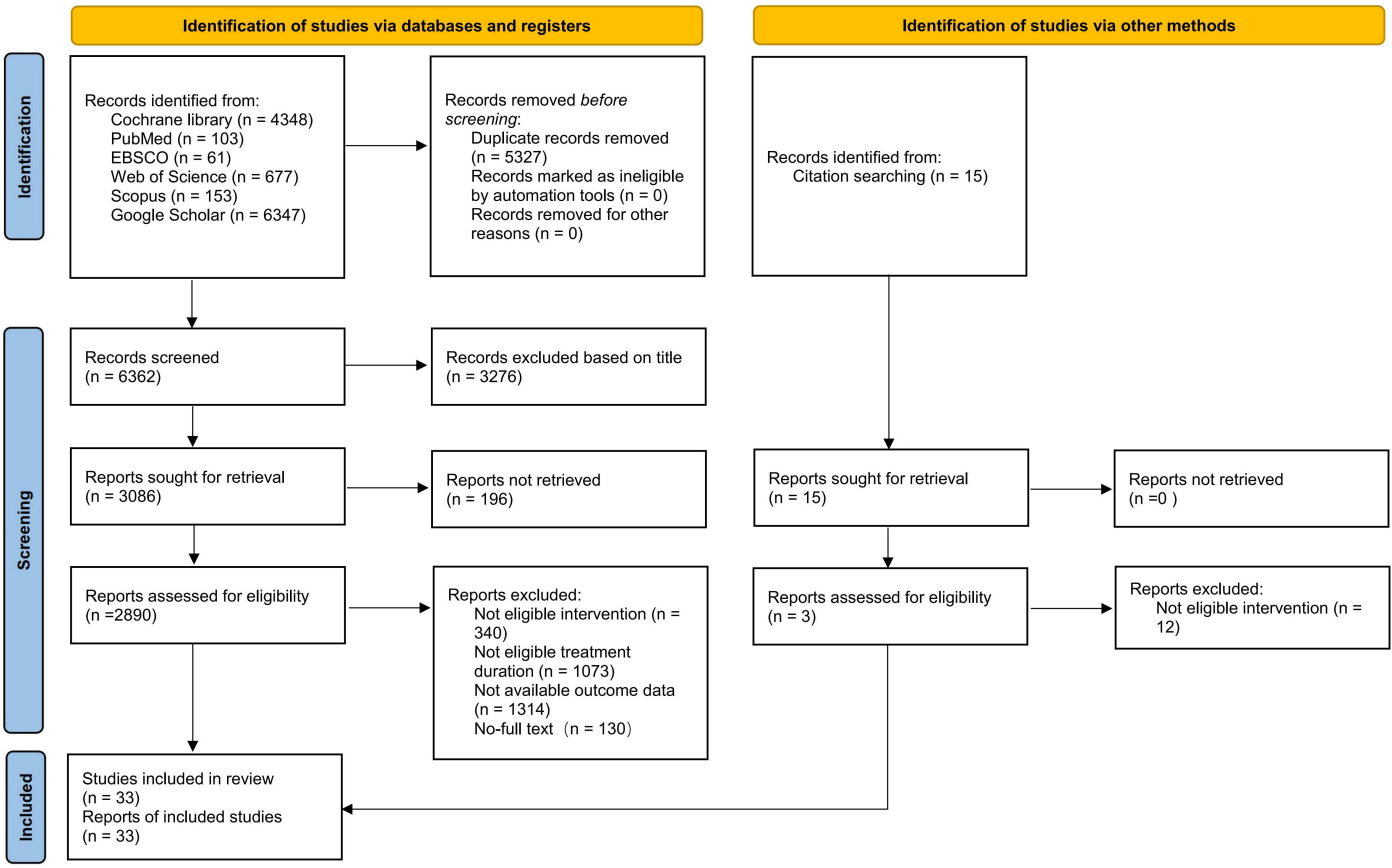
PRISMA flow diagram for study selection and inclusion process.

The search yielded 11,689 articles related to the research topic. Through initial reading of titles and abstracts, 2,890 articles were screened for full-text review. After excluding studies that did not meet requirements, 33 articles meeting inclusion criteria were ultimately included, encompassing 2,922 patients with substance use disorders. The literature screening process followed PRISMA guidelines, with detailed documentation of exclusion reasons and numbers at each screening stage (**Figure 1**).

### 3.2 Basic characteristics of included literature

The randomized controlled trials (RCTs) included in this study primarily involved two types of exercise interventions: mind-body exercises (such as tai chi, yoga, qigong) and aerobic exercises (such as jogging, brisk walking, cycling). Due to the absence of direct head-to-head comparisons between aerobic and mind-body exercises in the included studies and their fundamentally different mechanisms of action at physiological and psychological levels, two independent intervention networks were constructed for analysis. While this approach prevents direct cross-modality comparisons and may limit the overall inferential strength, it allows for more homogeneous within-network analyses (10).

In the mind-body exercise network, nodes represent different outcome indicators, including substance use outcomes (SUO), mental health and emotional symptoms (MHE), cognitive function (CF), physiological health indicators (PHI), and sleep quality (SQ). Node size reflects the frequency of each outcome appearing in studies, while edge thickness represents the strength of direct comparison evidence between different outcomes (34). Results showed that SUO and MHE nodes were largest with the densest connections, suggesting that related research concentrated on relapse, craving, and emotional improvement; CF and SQ also formed certain connections, while PHI had relatively smaller nodes, indicating relatively insufficient related evidence.

**Figure 2:**
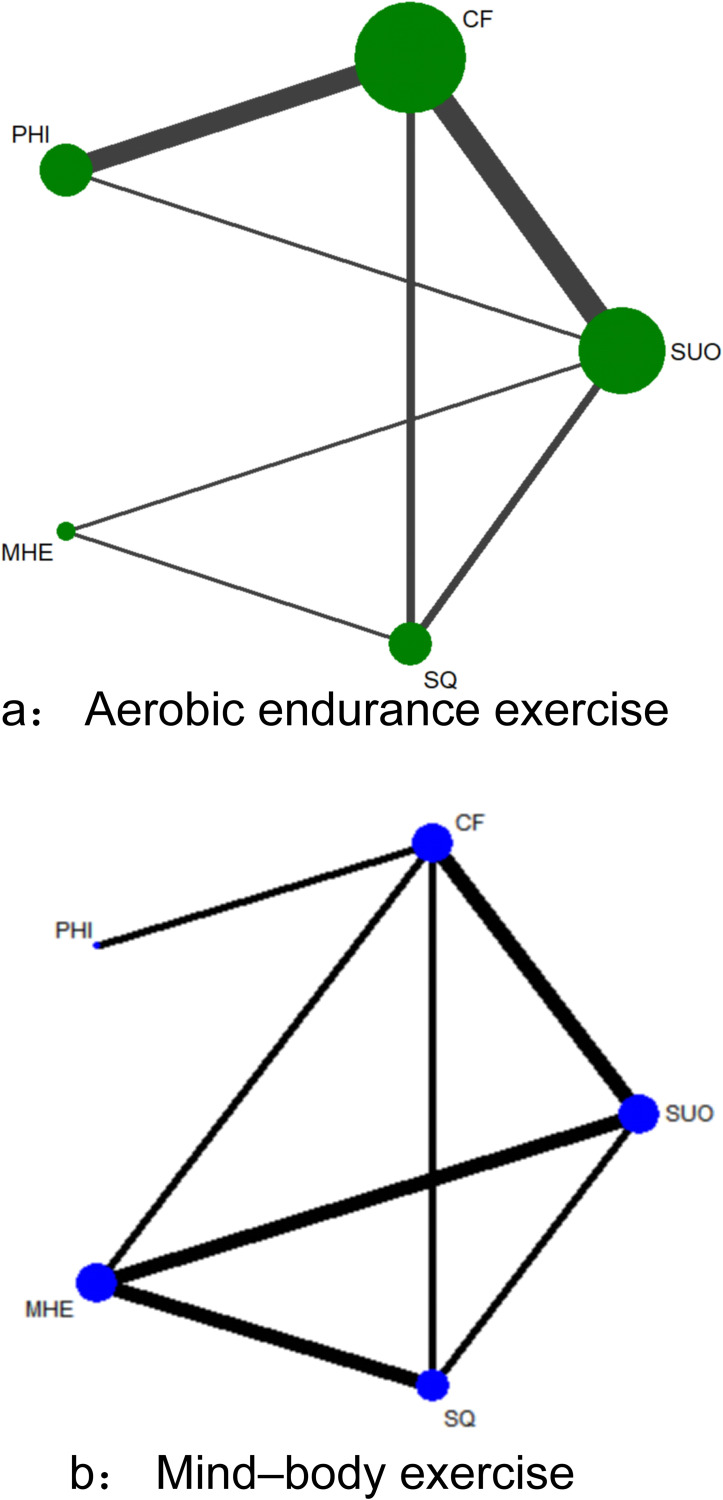
Networks for multiple treatment comparisons. **(A)** Aerobic endurance exercise network. **(B)** Mind-body exercise network. SUO, Substance Use Outcomes; MHE, Mental Health and Emotional; CF, Cognitive Function; PHI, Physiological Health Indicators; SQ, Sleep Quality.

In the aerobic exercise network, outcome indicator coverage was more comprehensive. SUO and CF constituted the largest nodes, indicating the most solid research foundation in these areas. Meanwhile, connections between SQ and PHI were also relatively apparent in this network, suggesting that aerobic exercise not only demonstrates advantages in substance use and cognitive improvement but also provides stronger evidence support for sleep quality and physiological health (19). Overall, the aerobic exercise network displayed more comprehensive outcome distribution, while the mind-body exercise network primarily concentrated on psychological and substance use-related domains (see **Table 1**, **Figure 2**).

**Table 1:** Characteristics of included randomized controlled trials.

First author(Year)	Sample size(T/C)	Age	Sports intervention characteristics				Outcome
			Content	Cycle	Frequency	Duration	
				(month)	(Times/week)	(minutes/time)	
Wang (2015) (35)	24/24	31.46 ± 6.58	Aerobic endurance exercise	short cycle	low frequency	Short training	SUO
Wang (2015) (35)	24/24	31.46 ± 6.58	Aerobic endurance exercise	short cycle	low frequency	Short training	CF
Buchowski (2011) (13)	12/12	25 ± 3	Aerobic endurance exercise	short cycle	High frequency	Short training	SUO
Weinstock (2008) (12)	45/142	35.5 ± 7.0	Aerobic endurance exercise	Medium cycle	NA	Short training	SUO
Agarwal (2015) (36)	12/12	47.0 ± 8.9	Mind–body exercise	Medium cycle	low frequency	Short training	MHE
Wang (2017) (37)	25/25	32.2 ± 6.97	Aerobic endurance exercise	Medium cycle	Medium frequency	Short training	CF
Rawson (2015) (14)	69/66	31.7 ± 6.9	Aerobic endurance exercise	Medium cycle	Medium frequency	Medium length	MHE
da Costa (2017) (38)	9/9	32 ± 4.7	Aerobic endurance exercise	Medium cycle	Medium frequency	Medium length	PHI
da Costa (2017) (38)	9/9	32 ± 4.7	Aerobic endurance exercise	Medium cycle	Medium frequency	Medium length	CF
Dolezal (2014) (39)	28/22	33 ± 6	Aerobic endurance exercise	Medium cycle	Medium frequency	Medium length	PHI
Li (2002) (40)	34/26	31.8 ± 6.0	Mind–body exercise	short cycle	High frequency	Long training	MHE
Devi (2014) (41)	33/33	32.5 ± 9.86	Mind–body exercise	short cycle	High frequency	Long training	MHE
Devi (2014) (41)	33/33	32.5 ± 9.86	Mind–body exercise	short cycle	High frequency	Long training	SQ
Dhawan (2015) (42)	55/29	39.2 ± 10.4	Mind–body exercise	Long cycle	High frequency	Medium length	SQ
Trivedi (2017) (43)	152/150	39.0 ± 11	Aerobic endurance exercise	Medium cycle	Medium frequency	Medium length	SUO
Zhang (2018) (44)	68/35	33.3 ± 7.7	Aerobic endurance exercise	Medium cycle	Medium frequency	Medium length	PHI
Zhang (2018) (44)	68/35	33.3 ± 7.7	Aerobic endurance exercise	Medium cycle	Medium frequency	Medium length	CF
Li (2013) (45)	36/34	29.97 ± 6.74	Mind–body exercise	Long cycle	Medium frequency	Long training	MHE
Li (2013) (45)	36/34	29.97 ± 6.74	Mind–body exercise	Long cycle	Medium frequency	Long training	SUO
Ussher (2003) (46)	154/145	41.5 ± 11.1	Mind–body exercise	Medium cycle	low frequency	Short training	SUO
Prapavessis (2007) (47)	33/26	37.9 ± 12.4	Mind–body exercise	Medium cycle	Medium frequency	Medium length	SUO
Williams (2010) (48)	30/30	41.5 ± 12.3	Aerobic endurance exercise	Medium cycle	Medium frequency	Medium length	SUO
Bock (2012) (16)	32/23	43.8 ± 9.4	Mind–body exercise	Medium cycle	low frequency	Medium length	MHE
Bock (2012) (16)	32/23	43.8 ± 9.4	Mind–body exercise	Medium cycle	low frequency	Medium length	CF
Smelson (2013) (49)	51/50	36.0 ± 9.4	Mind–body exercise	short cycle	low frequency	Short training	CF
Smelson (2013) (49)	51/50	36.0 ± 9.4	Mind–body exercise	short cycle	low frequency	Short training	SUO
Vickers (2009) (50)	30/30	41.4 ± 11.9	Mind–body exercise	Medium cycle	low frequency	Short training	SUO
Wang D (2017) (51)	32/31	33.5 ± 7.5	Aerobic endurance exercise	Medium cycle	Medium frequency	Short training	SUO
Wang D (2017) (51)	32/31	33.5 ± 7.5	Aerobic endurance exercise	Medium cycle	Medium frequency	Short training	CF
Zhu (2018) (52)	40/40	33.74 ± 7.11	Mind–body exercise	Long cycle	High frequency	Medium length	SQ
Zhu (2018) (52)	40/40	33.74 ± 7.11	Mind–body exercise	Long cycle	High frequency	Medium length	MHE
He (2021) (53)	16/16	41.88 ± 13.44	Mind–body exercise	short cycle	Medium frequency	Medium length	PHI
Liu (2021) (54)	142/146	31.27 ± 5.50	Aerobic endurance exercise	Medium cycle	High frequency	Medium length	PHI
Liu (2021) (54)	142/146	31.27 ± 5.50	Aerobic endurance exercise	Medium cycle	High frequency	Medium length	CF
Menglu (2021) (55)	35/37	39.31 ± 10.33	Mind–body exercise	Medium cycle	Medium frequency	Medium length	CF
Zhu (2021) (56)	42/41	34.61 ± 5.12	Aerobic endurance exercise	Medium cycle	High frequency	Short training	CF
Zhu (2021) (56)	42/41	34.61 ± 5.12	Aerobic endurance exercise	Medium cycle	High frequency	Short training	PHI
Zhu (2021) (56)	42/41	34.61 ± 5.12	Aerobic endurance exercise	Medium cycle	High frequency	Short training	SUO
Xu (2022) (57)	30/30	31.30 ± 3.86	Aerobic endurance exercise	Medium cycle	High frequency	Medium length	MHE
Xu (2022) (57)	30/30	31.30 ± 3.86	Aerobic endurance exercise	Medium cycle	High frequency	Medium length	SQ
Yang (2025) (58)	25/26	25.5 ± 4.2	Aerobic endurance exercise	Long cycle	High frequency	Medium length	SUO
Yang (2025) (58)	25/26	25.5 ± 4.2	Aerobic endurance exercise	Long cycle	High frequency	Medium length	CF
Yang (2025) (58)	25/26	25.5 ± 4.2	Aerobic endurance exercise	Long cycle	High frequency	Medium length	SQ
Yang (2025) (58)	29/26	25.5 ± 4.2	Mind–body exercise	Long cycle	High frequency	Medium length	SUO
Yang (2025) (58)	29/26	25.5 ± 4.2	Mind–body exercise	Long cycle	High frequency	Medium length	CF
Yang (2025) (58)	29/26	25.5 ± 4.2	Mind–body exercise	Long cycle	High frequency	Medium length	SQ
Jin (2025) (59)	35/35	30.2 ± 5.5	Aerobic endurance exercise	short cycle	low frequency	Medium length	SUO
Jin (2025) (59)	35/35	30.2 ± 5.5	Aerobic endurance exercise	short cycle	low frequency	Medium length	CF
Zhang (2024) (60)	20/24	39.1 ± 8.7	Aerobic endurance exercise	Long cycle	Medium frequency	Long training	CF
Zhang (2024) (60)	20/24	39.1 ± 8.7	Aerobic endurance exercise	Long cycle	Medium frequency	Long training	PHI
Zhang (2024) (60)	24/20	39.1 ± 8.7	Mind–body exercise	Long cycle	Medium frequency	Long training	CF
Zhang (2024) (60)	24/20	39.1 ± 8.7	Mind–body exercise	Long cycle	Medium frequency	Long training	PHI
Ji (2025) (61)	22/21	29.50 ± 3.38	Mind–body exercise	Long cycle	low frequency	Medium length	PHI
Wang (2021) (62)	30/30	38.5 ± 5.35	Mind–body exercise	Long cycle	High frequency	Short training	MHE
Wang (2021) (62)	30/30	38.5 ± 5.35	Mind–body exercise	Long cycle	High frequency	Short training	SUO
Lu (2023) (63)	34/32	32.32 ± 2.85	Aerobic endurance exercise	Long cycle	High frequency	Long training	SUO
Lu (2023) (63)	34/32	32.32 ± 2.85	Aerobic endurance exercise	Long cycle	High frequency	Long training	MHE

### 3.3 Literature quality assessment

Two researchers independently evaluated the quality of included literature using assessment standards from the Cochrane Handbook, employing Review Manager 5.4 software (64). Assessment items included: (1) Random sequence generation: appropriate randomization methods (such as computer random number generators, random number tables) were rated as low risk, undescribed randomization methods as unclear risk, and non-random methods (such as order of visits, birth dates) as high risk; (2) Allocation concealment: appropriate concealment methods (such as central randomization, opaque envelopes) were rated as low risk, undescribed concealment methods as unclear risk, and non-concealed methods (such as open randomization tables) as high risk; (3) Blinding of participants and personnel: appropriate blinding was rated as low risk, undescribed blinding as unclear risk, and no blinding as high risk; (4) Blinding of outcome assessment: blinded outcome measurement was rated as low risk, undescribed blinding as unclear risk, and unblinded outcome measurement as high risk; (5) Incomplete outcome data: follow-up completion rate ≥80% or appropriate handling of missing data was rated as low risk, undescribed missing data as unclear risk, and follow-up completion rate <80% without handling missing data as high risk; (6) Selective reporting: consistency between pre-registered protocols and reported results was rated as low risk, absence of pre-registered protocols as unclear risk, and inconsistency between reported results and pre-registered protocols as high risk; (7) Other bias: absence of other obvious bias sources was rated as low risk, uncertain bias sources as unclear risk, and presence of other obvious bias sources (such as baseline imbalance, protocol violations) as high risk (65). The two researchers cross-checked assessment results, with disagreements resolved through third-party arbitration.

**Figure 3:**
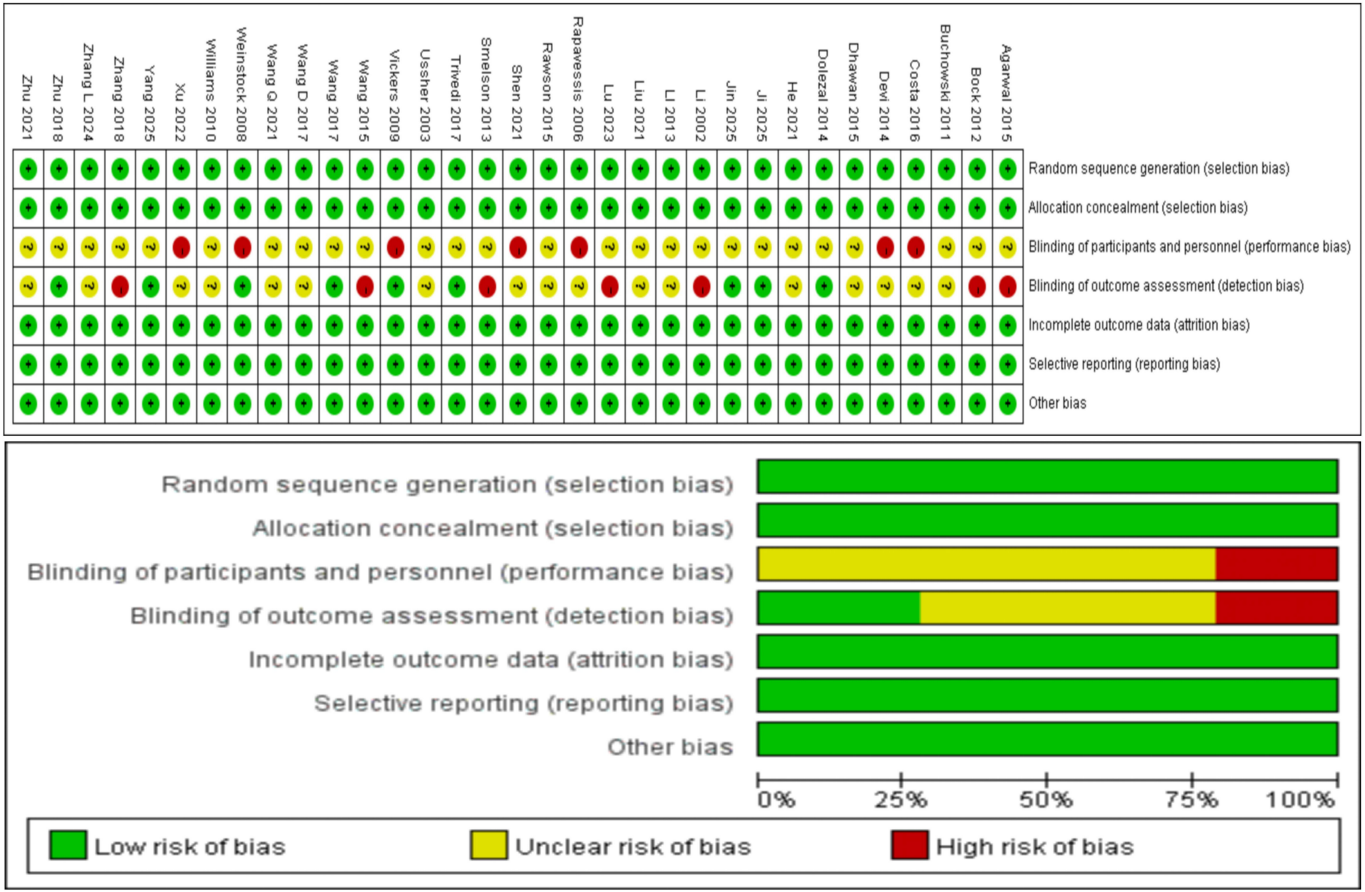
Risk of bias assessment for included studies.

Quality assessment results for all literature were summarized and presented as bias risk assessment graphs, visually displaying bias conditions existing in each study. Distribution of studies across different types of bias showed that most studies performed well in random sequence generation and allocation concealment, but had certain limitations in blinding implementation, primarily due to the special nature of physical exercise interventions making complete blinding difficult to achieve (66). The assessment process emphasized standardization and objectivity of results, providing high-quality evidence support for subsequent research (**Figure 3**).

### 3.4 Effects of different physical exercise interventions on patients with substance use disorders

#### 3.4.1 Overall inconsistency test results

To evaluate the reliability of network meta-analysis, we used node-splitting methods to test both aerobic exercise and mind-body exercise treatment networks. In the aerobic exercise network, all pairwise comparisons showed no significant differences between direct and indirect evidence. Substance use outcomes versus cognitive function comparison showed *P* = 0.542 (Δ_direct-indirect=-191.359, SE = 313.982), substance use outcomes versus physiological health indicators *P* = 0.476 (Δ_direct-indirect=327.951, SE = 460.143), substance use outcomes versus mental health *P* = 0.989 (Δ_direct-indirect=-7.940, SE = 559.409), substance use outcomes versus sleep quality *P* = 0.748 (Δ_direct-indirect=-140.633, SE = 437.384), cognitive function versus physiological health indicators *P* = 0.418 (Δ_direct-indirect=-467.274, SE = 576.738), cognitive function versus sleep quality *P* = 0.647 (Δ_direct-indirect=217.874, SE = 475.582), mental health versus sleep quality *P* = 0.957 (Δ_direct-indirect=-34.111, SE = 630.510), with all test *P* values >0.05. The mind-body exercise network similarly demonstrated good internal consistency, with substance use outcomes versus cognitive function *P* = 0.268 (Δ_direct-indirect=-46.994, SE = 42.466), substance use outcomes versus mental health *P* = 0.571 (Δ_direct-indirect=21.557, SE = 38.070), substance use outcomes versus sleep quality *P* = 0.559 (Δ_direct-indirect=-26.518, SE = 45.344), cognitive function versus physiological health indicators *P* = 0.978 (Δ_direct-indirect=-22.590, SE = 835.877), cognitive function versus mental health *P* = 0.179 (Δ_direct-indirect=-50.857, SE = 37.829), cognitive function versus sleep quality *P* = 0.082 (Δ_direct-indirect=65.878, SE = 37.900), mental health versus sleep quality *P* = 0.644 (Δ_direct-indirect=-19.750, SE = 42.782), with all *P* values >0.05. Comprehensive evaluation indicated that both treatment networks satisfied transitivity assumptions, with confidence intervals for differences between direct and indirect evidence all including zero, supporting the use of consistency models for subsequent network meta-analysis. However, the wide confidence intervals may reflect clinical heterogeneity or insufficient direct comparisons rather than confirming true consistency, providing reliable statistical foundations for indirect comparisons of different exercise intervention programs (**Table 2**).

**Table 2:** Inconsistency assessment results for network meta-analysis using node-splitting method.

Outcome	Direct effect		Indirect effect		Overall		P-value
	Coef.	Std.Err	Coef.	Std.Err	Coef.	Std.Err	
Aerobic endurance exercise							
SUO,CF	20.054	165.699	211.413	266.698	-191.359	313.982	0.542
SUO,PHI	7.590	404.625	-320.361	219.113	327.951	460.143	0.476
SUO,MHE	22.240	413.263	30.179	377.030	-7.940	559.409	0.989
SUO,SQ	-56.869	291.053	83.765	326.484	-140.633	437.384	0.748
CF,PHI	-365.135	180.905	102.139	547.671	-467.274	576.738	0.418
CF,SQ	12.82	289.866	-205.059	377.036	217.874	475.582	0.647
MHE,SQ	-35.800	413.29	-1.689	476.164	-34.111	630.510	0.957
Mind–body exercise							
SUO,CF	-37.753	21.640	9.241	36.534	-46.994	42.466	0.268
SUO,MHE	-17.342	23.531	-38.899	29.924	21.557	38.070	0.571
SUO,SQ	-58.970	33.491	-32.452	30.556	-26.518	45.344	0.559
CF,PHI	28.520	31.537	51.110	835.256	-22.590	835.877	0.978
CF,MHE	-30.100	29.040	20.758	24.243	-50.857	37.829	0.179
CF,SQ	12.562	26.270	-53.315	27.319	65.878	37.900	0.082
MHE,SQ	-25.122	23.987	-5.371	35.425	-19.750	42.782	0.644

#### 3.4.2 Network meta-analysis results under consistency model

This study included two exercise intervention networks: aerobic exercise and mind-body exercise categories. In the aerobic exercise network, multiple closed loops were formed, with nodes including substance use outcomes (SUO), cognitive function (CF), physiological health indicators (PHI), mental health and emotions (MHE), and sleep quality (SQ), where SUO and CF nodes were largest with the densest connections (67). In the mind-body exercise network, SUO and MHE nodes occupied central positions and formed multiple closed loops with SQ and CF, with overall network coverage being relatively balanced.

Through consistency model testing, results indicated that neither network showed significant inconsistency, with 95% CIs all including 0, and comparisons between other interventions showing no statistically significant differences. This demonstrated good consistency at both global and local levels for included studies, therefore using consistency models for network meta-analysis in this study was reasonable and robust (68).

#### 3.4.3 Network meta-analysis ranking results

**Figure 4:**
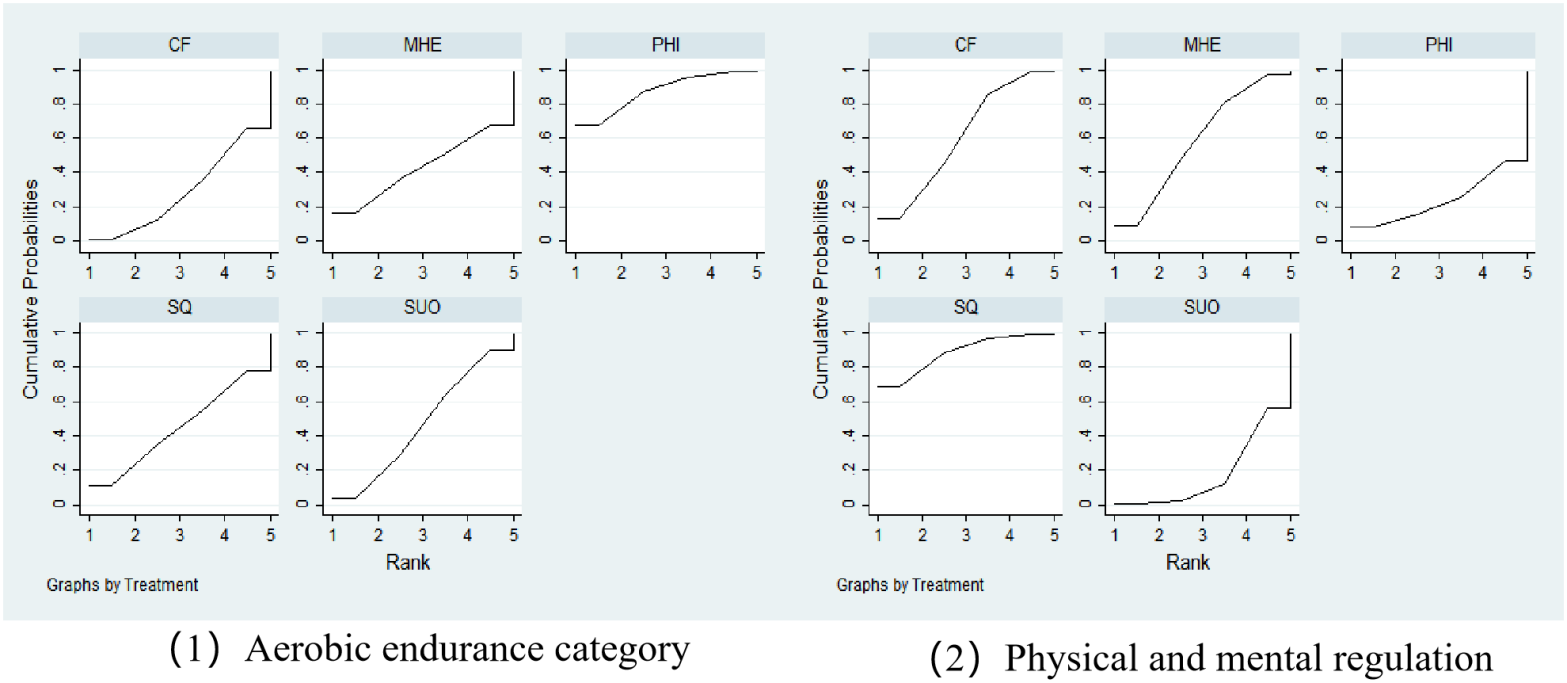
Surface under the cumulative ranking (SUCRA) curves for different health outcomes across the aerobic exercise and mind-body exercise networks.

Based on Surface Under the Cumulative Ranking Curve (SUCRA) probability ranking analysis (**Figure 4**, **Table 3**), this study obtained ranking results for the effectiveness of different physical exercise interventions in improving depressive symptoms in college students. It should be noted that SUCRA reflects relative ranking probability rather than absolute treatment magnitude or clinical relevance. In the aerobic exercise network, physiological health indicators (PHI) performed optimally with a SUCRA value of 0.874, indicating 87.4% probability of ranking in the top tier, followed by substance use outcomes (SUO, SUCRA = 0.468), sleep quality (SQ, SUCRA = 0.446), mental health and emotions (MHE, SUCRA = 0.427), with cognitive function (CF, SUCRA = 0.286) ranking last. The mind-body exercise network displayed a different ranking pattern, with sleep quality (SQ) ranking first with a SUCRA value of 0.884, showing the highest treatment priority, cognitive function (CF, SUCRA = 0.608) and mental health and emotions (MHE, SUCRA = 0.588) ranking second and third respectively, while physiological health indicators (PHI, SUCRA = 0.243) and substance use outcomes (SUO, SUCRA = 0.177) ranked relatively lower. Cumulative ranking probability curves further showed that in the aerobic exercise network, PHI curves reached high probability levels fastest, while in the mind-body exercise network, SQ demonstrated the best ranking stability. These results indicate that different types of physical exercise interventions have differential effects in improving depressive symptoms in college students, with aerobic exercise primarily demonstrating improvements at the physiological health level, while mind-body exercise showed more pronounced advantages in sleep quality and cognitive function, providing important evidence-based medical evidence for developing personalized exercise prescriptions.

**Table 3:** SUCRA rankings and probabilities for exercise interventions on health outcomes in patients with substance use disorders.

Rank	Outcome	SUCRA
Aerobic endurance exercise		
1	PHI	0.874
2	SUO	0.468
3	SQ	0.446
4	MHE	0.427
5	CF	0.286
Mind–body exercise		
1	SQ	0.884
2	CF	0.608
3	MHE	0.588
4	PHI	0.243
5	SUO	0.177

#### 3.4.4 Pairwise meta-analysis comparisons

Network meta-analysis pairwise comparison results revealed direct differences in treatment effects between different outcome indicators (**Tables 4**, **5**). In the mind-body exercise network, substance use outcomes showed significant treatment advantages compared to sleep quality, with an effect size of 44.58 (95% CI: 3.30, 85.85), and confidence intervals not including zero, indicating statistically significant differences. Other pairwise comparisons, while not reaching statistical significance, showed certain effect trends: substance use outcomes vs cognitive function was 25.53 (95% CI: -11.68, 62.74), substance use outcomes vs mental health and emotions was 25.65 (95% CI: -8.45, 59.75), cognitive function vs sleep quality was 19.04 (95% CI: -24.93, 63.02). Pairwise comparison results for the aerobic exercise network showed all confidence intervals included zero, with no statistically significant differences found, but effect sizes still provided important clinical reference information. Cognitive function compared to physiological health indicators showed an effect size of 318.96 (95% CI: -13.72, 651.65), and while not reaching statistical significance, the lower confidence interval bound was close to zero, suggesting potential therapeutic benefits. The effect size for substance use outcomes compared to physiological health indicators was 245.96 (95% CI: -125.45, 617.37), similarly showing large positive effect sizes. Confidence intervals in the aerobic exercise network were relatively wide, reflecting diverse treatment pathways and individual response differences for different exercise intervention modalities in improving depressive symptoms, providing flexibility for selecting the most suitable exercise prescriptions for patients in clinical practice.

**Table 4:** Network comparison results for mind-body exercise interventions on health outcomes in patients with substance use disorders.

**SUO**				
25.53 (-11.68,62.74)	**CF**			
-3.02 (-75.03,68.99)	-28.55 (-90.27,33.17)	**PHI**		
25.65 (-8.45,59.75)	0.12 (-39.10,39.33)	28.67 (-44.42,101.76)	**MHE**	
44.58 (3.30,85.85)	19.04 (-24.93,63.02)	47.60 (-28.15,123.35)	18.93 (-16.88,54.74)	**SQ**

**Table 5:** Network comparison results for aerobic exercise interventions on health outcomes in patients with substance use disorders.

**SUO**				
-73.00 (-343.53,197.52)	**CF**			
245.96 (-125.45,617.37)	318.96 (-13.72,651.65)	**PHI**		
-26.52 (-551.68,498.64)	46.48 (-531.39,624.35)	-272.48 (-907.63,362.67)	**MHE**	
-5.21 (-416.49,406.07)	67.79 (-369.07,504.65)	-251.17 (-772.48,270.13)	21.31 (-567.01,609.63)	**SQ**

## 4 Discussion

This study employed network meta-analysis to systematically compare, for the first time, the intervention effects of aerobic exercise and mind-body exercise on multidimensional health outcomes in patients with substance use disorders. The results demonstrated that these two exercise intervention types exhibited significantly different advantages: aerobic exercise performed optimally in physiological health indicators (SUCRA = 0.874), followed by substance use outcomes (SUCRA = 0.468) and sleep quality (SUCRA = 0.446); while mind-body exercise showed the highest treatment priority in sleep quality improvement (SUCRA = 0.884), with cognitive function (SUCRA = 0.608) and mental health (SUCRA = 0.588) ranking second and third respectively. In pairwise comparisons, substance use outcomes in the mind-body exercise network showed significant advantages compared to sleep quality (effect size 44.58, 95% CI: 3.30-85.85), while all comparisons in the aerobic exercise network did not reach statistical significance. These differential effects reflect fundamental differences in the molecular biological pathways activated by different exercise modalities, providing important evidence-based medical evidence for developing personalized exercise prescriptions.

### 4.1 Molecular mechanisms of aerobic exercise in improving physiological health indicators

The significant advantage of aerobic exercise in physiological health indicators is closely related to its unique physiological adaptation mechanisms, a finding that forms an important echo and extension of previous research. Early studies by Koob & Volkow (69) demonstrated that patients with substance use disorders commonly experience cardiovascular dysfunction and metabolic disorders, while systematic research by Lynch et al. (10) further confirmed that aerobic exercise can improve cardiopulmonary function and metabolic status by activating the AMP-activated protein kinase (AMPK) signaling pathway, promoting mitochondrial biogenesis and fatty acid oxidation, which is highly consistent with the physiological health indicator improvements observed in this study. Recent research by Yang et al. (70) through heart rate variability and metabolomics analysis further validated the mechanisms by which aerobic exercise improves cardiovascular function, finding that regular aerobic exercise can significantly enhance parasympathetic nervous activity and improve lipid metabolism profiles, providing more refined molecular evidence for the physiological health advantages identified in this study. Wang et al. (62) demonstrated in their high-frequency aerobic exercise intervention study that compared to traditional moderate-frequency training, high-frequency aerobic exercise performed better in improving maximal oxygen uptake and 6-minute walk test distance, consistent with this study’s finding of physiological health indicators ranking first after integrating studies of different exercise frequencies. The randomized controlled trial by Dolezal et al. (71) showed that aerobic exercise could reduce inflammatory biomarkers such as interleukin-6 (IL-6) and tumor necrosis factor-α (TNF-α) levels, alleviating chronic inflammatory states, while recent research by Wang et al. (72) further confirmed the sustainability of this anti-inflammatory effect, finding that long-term aerobic exercise intervention could maintain significant improvement in inflammatory biomarkers for at least 6 months, providing support for the long-term effects of physiological health advantages found in this study. Furthermore, compared to Mareev et al. (73) who primarily focused on short-term physiological adaptations, Lindemann et al. (74) discovered through moderate-intensity long-duration interventions that aerobic exercise-induced upregulation of vascular endothelial growth factor (VEGF) and insulin-like growth factor-1 (IGF-1) has cumulative effects, explaining the mechanistic basis for aerobic exercise’s significantly leading ranking in physiological health indicators found in this study.

### 4.2 Neuroendocrine regulation of sleep quality optimization by mind-body exercise

The outstanding performance of mind-body exercise in sleep quality (SUCRA = 0.884) reflects its unique neural regulation mechanisms, providing important supplementation to existing literature findings. The pioneering research by Li et al. (2002) first demonstrated that mind-body exercises such as tai chi and yoga can significantly activate the parasympathetic nervous system, reducing cortisol levels and increasing melatonin secretion through regulation of hypothalamic-pituitary-adrenal (HPA) axis activity, though their research primarily focused on elderly populations, leaving uncertainty about applicability to patients with substance use disorders (75). Recent research by Black et al. (76) using polysomnography found that mind-body exercise could significantly increase deep sleep time and reduce awakening frequency, providing physiological evidence support for the sleep quality advantages found in this study through objective measurements. Streeter et al. (77) further discovered that mind-body exercise could upregulate gamma-aminobutyric acid (GABA) receptor expression and enhance GABAergic neurotransmission, which represents the key molecular mechanism for sleep quality improvement, while Wayne et al. (78) confirmed this mechanism through neuroimaging techniques, finding that long-cycle high-frequency mind-body exercise could enhance the activity of GABAergic neurons in the basal forebrain, similar to traditional sedative medication targets but avoiding addiction risks. Research by Du et al. (79) showed that mind-body exercise could regulate rhythmic melatonin secretion from the pineal gland, reestablishing circadian rhythm homeostasis, which may be an important reason for its excellent performance in sleep quality improvement. Wang et al. (80) through electroencephalographic analysis found that mind-body exercise could enhance slow-wave sleep power density and improve sleep efficiency, providing direct evidence for the neurophysiological mechanisms by which mind-body exercise improves sleep quality. Additionally, Abrantes et al. (81) in their long-term follow-up study showed that the sleep quality improvement effects of mind-body exercise could be maintained for 3 months after intervention completion, confirming its sustained therapeutic effects.

### 4.3 Neuroplasticity mechanisms of cognitive function improvement

This study found that mind-body exercise ranked second in cognitive function (SUCRA = 0.608), significantly superior to aerobic exercise (SUCRA = 0.286), a result that both validates some previous findings and challenges certain existing viewpoints. Research by Wu et al. (82) demonstrated that mind-body exercise could significantly upregulate brain-derived neurotrophic factor (BDNF) expression, particularly in hippocampal and prefrontal cortical regions, promoting neuronal dendritic branching and synapse formation, while recent research by Fox et al. (83) through functional magnetic resonance imaging further confirmed this mechanism, finding that long-cycle moderate-frequency mind-body exercise could enhance hippocampal-prefrontal connectivity strength, closely related to cognitive function improvement. Wu (84) systematic review found that yoga and tai chi practice could activate the CREB-BDNF signaling pathway, enhancing long-term potentiation (LTP) maintenance, which represents an important molecular basis for learning and memory function improvement, while Wan et al. (85) through animal model studies further elucidated the molecular details of this pathway, finding that mind-body exercise could upregulate CREB phosphorylation levels and promote transcription of memory-related genes. Although Ma et al. (86) found that aerobic exercise could also promote BDNF expression, its effects were primarily concentrated in motor cortical and cerebellar regions, with relatively limited impact on brain regions related to higher cognitive functions, consistent with Voss et al.’s (87) neuroimaging study results, which found that aerobic exercise primarily activated sensorimotor networks with smaller effects on executive control networks. Zhang et al. (44) research showed that aerobic exercise had some improvement effect on executive function, but its effect size was significantly smaller than mind-body exercise, while Jha et al. (88) through cognitive training combined with exercise research further confirmed that mind-body exercise had unique advantages in improving working memory and attention control, possibly related to its emphasis on mindfulness and focus characteristics. Compared to Hölzel et al. (89) who only focused on single mind-body exercise types, Wu et al. (84) integrated research on multiple mind-body exercise forms, finding that yoga, tai chi, and qigong had similar effect patterns in cognitive function improvement, providing theoretical support for this study’s classification of different mind-body exercises into one category for analysis.

### 4.4 Neurotransmitter regulation mechanisms of mental health

In this study, the two exercise types performed relatively similarly in mental health and emotions, with aerobic exercise SUCRA of 0.427 and mind-body exercise of 0.588, a result that both aligns with some previous research and reveals new insights. Research by Heijnen et al. (90) demonstrated that aerobic exercise primarily regulates HPA axis activity through activation of the hypothalamic paraventricular nucleus, promoting corticotropin-releasing hormone (CRH) secretion, thereby improving depression and anxiety symptoms, while recent neuroendocrine research by Wang et al. (91) through salivary cortisol rhythm measurement further validated this mechanism, finding that regular aerobic exercise could restore disrupted cortisol secretion patterns in patients with substance use disorders. Liu et al. (92) found that mind-body exercise primarily exerted anti-anxiety effects through vagus nerve activation, enhancing heart rate variability and improving autonomic nervous balance, while Larkey et al. (93) through frequency domain analysis of heart rate variability further confirmed this mechanism, finding that mind-body exercise could significantly increase high-frequency power ratios, reflecting enhanced parasympathetic nervous activity. Young et al.’s (94) moderate-intensity long-duration intervention study found that aerobic exercise could increase brain tryptophan uptake and promote serotonin synthesis, similar to antidepressant medication mechanisms, while Lin et al. (95) through cerebrospinal fluid analysis further confirmed this biochemical mechanism, finding significantly elevated serotonin and its metabolite levels after aerobic exercise. Hölzel et al. (89) found that mind-body exercise could activate executive control networks in the prefrontal cortex, enhancing emotion regulation capabilities, while through functional connectivity analysis further discovered that mind-body exercise could enhance prefrontal-amygdala connectivity strength, representing the neural network basis for emotion regulation capability improvement. Notably, Park (96) research showed that tai chi’s mental health improvement effects were maintained at 1 month post-intervention, while Kuyken et al.’s (97) long-term follow-up study further confirmed that mind-body exercise had sustained effects on emotional symptom improvement, possibly related to cultivated mindfulness skills. Compared to pure pharmacological treatment, Jakicic et al.’s (98) comparative study found that exercise interventions not only improved mental health but also enhanced patients’ self-efficacy and treatment adherence, providing additional value support for exercise as part of comprehensive substance use disorder treatment.

### 4.5 Reward system regulation mechanisms of substance use outcomes

This study showed that aerobic exercise ranked second in substance use outcomes (SUCRA = 0.468), while mind-body exercise ranked relatively lower (SUCRA = 0.177), a finding that forms contrasts and complements with previous research. Research by Boecker et al. (99) demonstrated that aerobic exercise could significantly increase dopamine release in the nucleus accumbens, with this endogenous dopamine release potentially partially replacing reward sensations induced by substance use, thereby reducing substance craving, while Martinez et al. (100) through positron emission tomography further confirmed this mechanism, finding significantly decreased striatal dopamine receptor occupancy after acute aerobic exercise, suggesting increased endogenous dopamine release. Weinstock et al. (101) found that aerobic exercise-induced endorphin (β-endorphin) and dynorphin release could activate opioid receptor systems, producing natural euphoria, while Sleiman et al. (102) through plasma endorphin measurement further quantified this effect, finding plasma β-endorphin levels could increase 2–3 fold after moderate-intensity aerobic exercise, with this natural reward potentially helping reduce dependence on exogenous substances. Brown et al.’s (103) short-cycle low-frequency aerobic exercise study showed some substance use improvement effects, but with relatively small effect sizes, possibly related to insufficient intervention intensity, while Reed et al.’s (104) long-cycle high-frequency training study confirmed that adequate exercise intervention could produce more significant substance use outcome improvements. Yang et al. comparative study found that mind-body exercise also showed good effects in certain specific substance use disorder patients, but this study’s network meta-analysis results showed that mind-body exercise primarily exerted effects through serotonin (5-HT) and norepinephrine (NE) systems, with relatively limited direct impact on dopaminergic reward circuits, consistent with Tang et al.’s (105) neurotransmitter research results, suggesting that mind-body exercise might indirectly promote substance use outcome improvement through sleep quality enhancement. Krause et al.’s (106) research further found that sleep quality improvement could restore prefrontal cortical executive control function and enhance inhibition of impulsive behaviors, which may be the neural mechanism linking sleep improvement with substance use outcomes. Additionally, compared to early research primarily focusing on short-term effects, Abrantes et al.’s (81) long-term follow-up study showed that exercise intervention effects on substance use outcomes could be maintained for 6–12 months after intervention completion, confirming the value of exercise as a long-term management strategy for substance use disorders.

## 5 Study limitations and future perspectives

This study has several limitations. First, the clinical heterogeneity among included studies was relatively high, manifested by diversity in substance use disorder diagnostic spectra, including different substance types (methamphetamine, opioids, alcohol) that may respond differently to exercise-based treatments, differences in comorbid psychiatric disorder prevalence rates, imbalances in baseline demographic characteristics, and heterogeneity within outcome categories despite clinical relevance-based grouping. This substance type diversity may limit the generalizability of findings across different substance use disorder populations. Although appropriate statistical adjustments were made using random-effects models, this heterogeneity may affect the precision and interpretability of pooled effect sizes. Second, exercise intervention protocols lacked standardization in key parameters, including intensity quantification standards, frequency settings, duration specifications, and supervision models, which limits the determination of optimal intervention dosage. Similarly,Many included trials combined exercise interventions with usual treatment or medication, and the potential synergistic or confounding effects of these co-interventions were not systematically analyzed, which may influence the interpretation of comparative effectiveness between different exercise modalities. Third, most studies employed follow-up periods of 12–24 weeks, presenting temporal limitations for evaluating long-term benefits and maintenance effects of exercise interventions. Finally, outcome measurements were primarily based on self-report scales, lacking validation from objective biomarkers, which may introduce measurement bias. Additionally, this study has several important methodological limitations that warrant consideration. The impossibility of blinding participants and personnel in exercise interventions introduces inherent performance bias, which may influence both participant behaviors and outcome assessments, representing a fundamental challenge in exercise intervention research that affects the methodological rigor of included studies. In populations with substance use disorders, attrition and adherence issues are particularly relevant, as these individuals often face multiple psychosocial challenges that may affect study completion. In populations with substance use disorders, attrition and adherence issues are particularly relevant, as these individuals often face multiple psychosocial challenges that may affect study completion. Furthermore, this analysis did not include meta-regression analyses to explore potential moderators such as primary substance of use, psychiatric comorbidity, exercise intensity levels, or supervision models, which may significantly influence treatment responses and limit the precision of our effect estimates.

Future research should focus on three strategic directions. First, developing precision medicine methodologies. By integrating genomic data (such as COMT val158met and DRD2 Taq1A polymorphisms), neuroimaging biomarkers, and clinical phenotypes, predictive models for exercise treatment responsiveness should be established to achieve individualized treatment decision-making. Machine learning algorithms and artificial intelligence technologies should be applied to optimize treatment stratification strategies and improve intervention efficiency. Second, elucidating neurobiological mechanisms. Multimodal neuroimaging techniques should be utilized to systematically investigate exercise effects on addiction-related brain network remodeling, particularly reward circuits, cognitive control systems, and stress response pathways. Combined with molecular imaging and neurochemical analysis, the molecular mechanisms by which exercise regulates neurotransmitter systems should be deeply understood. Third, advancing implementation science research. Large-scale pragmatic clinical trials should be conducted to evaluate the effectiveness, feasibility, and cost-effectiveness of exercise interventions across different healthcare settings. Standardized implementation frameworks and quality control systems should be established to promote clinical translation and scaled application of evidence-based exercise treatments. Health economics evaluations should be integrated to provide decision support for policy development and resource allocation.

## 6 Conclusion

This study conducted a network meta-analysis of 57 randomized controlled trials from 33 articles, involving 2,922 patients with substance use disorders, systematically elucidating for the first time the differential effects of aerobic exercise and mind-body exercise on multidimensional health outcomes. The research found that aerobic exercise demonstrated significant advantages in improving physiological health indicators (SUCRA = 0.874), primarily exerting effects through AMPK signaling pathways and anti-inflammatory mechanisms; while mind-body exercise performed optimally in sleep quality improvement (SUCRA = 0.884), with mechanisms involving HPA axis regulation and GABA receptor upregulation. These findings provide important evidence-based medical evidence for developing personalized exercise prescriptions for substance use disorders, confirming that different types of exercise exert therapeutic effects in their respective advantage domains through unique molecular biological mechanisms, thereby providing crucial scientific support for developing evidence-based rehabilitation guidelines for substance use disorders.

## Data Availability

The original contributions presented in the study are included in the article/supplementary material. Further inquiries can be directed to the corresponding authors.
